# Infantile Spinal Atrophic Paralysis, and Other Cases

**Published:** 1892-12

**Authors:** T. M. Dillingham

**Affiliations:** New York, N. Y.


*Third Day—Morning Session.*

Thursday, June 23d, 10 A. M.

(Bureau of Surgery Continued.)

### INFANTILE SPINAL ATROPHIC PARALYSIS, AND OTHER CASES.

T. M. Dillingham, M. D., New York, N. Y.

Among the most important and trying cases brought to a practitioner for treatment is that of infantile spinal paralysis.

In the etiology of the old school there are no predisposing causes known, although mention is made of syphilitic infection and rheumatism as likely to develop a liability to it.

The more I see of such cases, in fact, of disease in any form, the greater my admiration of Samuel Hahnemann and my respect for his theory of disease. In the first volume of his *Chronic Diseases* can be found, and there only, a rational explanation of the cause of all serious ailments of living creatures. If I could be entirely freed from all the inherited diseases that are in me, I believe I would be safe from every acute disease known to the world of medicine, except syphilis and gonorrhœa.

Here is this ailment called infantile spinal paralysis, which universally occurs in apparently well children. We have a perfect picture of the horrors of inheritance, and one ever to hold before the gaze of those who are dull in seeing the great truth in Hahnemann's explanation of chronic disease.

B. A., age five, as bright and lovely a boy as ever lived, was taken with high fever, after his usual play, among most perfect surroundings. He complains of pain in the head; would not allow them to move him from the position he had chosen, on his back, and if they did, screaming until he was again in the same position. The hands trembled and arms and legs twitched so that I feared spasms. He was very irritable, not the least his nature, and averse to light.

This fever continued for the greater part of four days, with but little remission, during which time he passed water very seldom, going twenty-four hours twice, although the bladder was full. There was no movement of the bowels for nearly two weeks (except on the seventh day, from an enema) (for the benefit of the family only). On the fifth day it was found he could not move his legs nor stand on them for an instant.

He was now happy again, had a good appetite and well, except for the paralysis.

The bladder gradually resumed its functions, and later the bowels. Then the left leg rapidly came to life, but the right one was useless.

After a while the child sat up in bed, surrounded by pillows; later the pillows were removed, but there was not yet strength enough in the right leg to keep him from pitching head foremost in almost any direction, if he lost his balance.

Finally he got upon his knees and a little later began to creep.

Then he began to climb up to chairs and like an infant learned to walk. So few of the muscles of the leg were active, however, that he could not walk without holding on to something, for months, and then in a frightfully distorted position of his little spine.

Now, what are we to do with such a case? The boy seemed well, and was apparently well in every particular, since the fever left him, except the paralysis.

We know the cause, although the parents are both strong and well, and there is no history of venereal disease of any kind. Several members of the family have had psoric conditions of the most aggravated form, especially the grandparents.

If it is not psoric, what is it? What else can commit such a crime?

I selected one or two remedies with great care, and used daily massage by a trained masseur.

As soon as he could stand up to a chair he was put into the wheel crutch, invented by Mr. S. G. Darrach, of Newark, and exercised an hour and a half, twice daily.

For several weeks there was no shrinking of the muscles, but later it showed itself markedly.

As soon as he could walk without help I put him into a raw-hide corset which kept his spine in the natural position as much as was possible, but it takes constant care to keep it there, as he is in almost constant motion, and although improving greatly in his ability to walk, some of the muscles yet refuse to awaken to their responsibility.

Practically all the deformity we see on the street, except that of maimed limbs, is the result of this disease, and to be able to cure the psoric lesion in the spinal cord quickly, and in the mean-time skillfully to preserve the symmetry of the body—would be a triumph. These cases usually pass into the hands of orthœpedic surgeons, so that their offices are crowded daily with the cases they have under charge. An interesting feature of the case was a consultation with one of these gentlemen, at the urgent request of the allopathic end of the family.

The surgeon agreed with me as to the diägnosis, but advised painting the spine with Iodine, giving palpable doses of Bell. and Strychnine and the daily use of electricity and massage.

The parents of the child are thoroughly Hahnemannian in their ideas, and the case was left in my care. The surgeon was free to admit that, considering the history of the acute attack, the case had made unusual progress toward recovery.

There remains much to do for this case yet, although the rotundity of the paralyzed leg is returning, and the boy is able to do more each week. I am by no means sure that it is not fatty infiltration instead of muscular development that is improving the appearance of the limb.

The satisfaction I have thus far derived from this case is, *first*, I feel sure as to the cause, and as to how the cause should be treated. Therefore, no experiment.

*Secondly*, by resorting to mechanical manipulation and supports I am preserving the natural form of the body until such time as it can be brought back to life, as it can be most quickly and safely done by the homœopathic remedy.

A second case of this kind came into my hands about the same time.

The primary attack was much lighter, and thought to be la grippe, followed by rheumatism.

Fortunately he had the best of care during the acute stage by a thorough Hahnemannian.

When I saw the child he walked as if his leg was asleep, and he appeared to "kick it out" when he exerted it.

No symptom above the knee of paralysis, but the ligaments about the knee were so much relaxed that I had to procure a brace to prevent hyper-extension.

He will also require further apparatus on the foot as the extensor muscles are yet inactive and the toe is drawn under, and without the proper support would result in club foot (Talipes equinus).

This case has received massage only.

I regret taking so much of your valuable time, but the observations of the pathologists on this lamentable ailment of children are so complete as regards its natural and pathological history that I will quote briefly from them.*

*Since writing the above, four more cases of Infantile Spinal Paralysis have come into my hands, two of them *neglected cases.*

